# Urinary Amine and Organic Acid Metabolites Evaluated as Markers for Childhood Aggression: The ACTION Biomarker Study

**DOI:** 10.3389/fpsyt.2020.00165

**Published:** 2020-03-31

**Authors:** Fiona A. Hagenbeek, Peter J. Roetman, René Pool, Cornelis Kluft, Amy C. Harms, Jenny van Dongen, Olivier F. Colins, Simone Talens, Catharina E. M. van Beijsterveldt, Marjolein M. L. J. Z. Vandenbosch, Eveline L. de Zeeuw, Sébastien Déjean, Vassilios Fanos, Erik A. Ehli, Gareth E. Davies, Jouke Jan Hottenga, Thomas Hankemeier, Meike Bartels, Robert R. J. M. Vermeiren, Dorret I. Boomsma

**Affiliations:** ^1^Department of Biological Psychology, Vrije Universiteit Amsterdam, Amsterdam, Netherlands; ^2^Amsterdam Public Health Research Institute, Amsterdam, Netherlands; ^3^Curium-LUMC, Department of Child and Adolescent Psychiatry, Leiden University Medical Center, Leiden, Netherlands; ^4^Good Biomarker Sciences, Leiden, Netherlands; ^5^Division of Analytical Biosciences, Leiden Academic Center for Drug Research, Leiden University, Leiden, Netherlands; ^6^The Netherlands Metabolomics Centre, Leiden, Netherlands; ^7^Department Special Needs Education, Ghent University, Ghent, Belgium; ^8^Toulouse Mathematics Institute, University of Toulouse, CNRS, Toulouse, France; ^9^Department of Surgical Sciences, University of Cagliari and Neonatal Intensive Care Unit, Cagliari, Italy; ^10^Avera Institute for Human Genetics, Sioux Falls, SD, United States; ^11^Amsterdam Neuroscience, Amsterdam, Netherlands

**Keywords:** metabolomics, childhood aggression, biomarkers, amines, organic acids, metabolites, neurotransmitters, oxidative stress

## Abstract

Biomarkers are of interest as potential diagnostic and predictive instruments in personalized medicine. We present the first urinary metabolomics biomarker study of childhood aggression. We aim to examine the association of urinary metabolites and neurotransmitter ratios involved in key metabolic and neurotransmitter pathways in a large cohort of twins (*N* = 1,347) and clinic-referred children (*N* = 183) with an average age of 9.7 years. This study is part of ACTION (Aggression in Children: Unraveling gene-environment interplay to inform Treatment and InterventiON strategies), in which we developed a standardized protocol for large-scale collection of urine samples in children. Our analytical design consisted of three phases: a discovery phase in twins scoring low or high on aggression (*N* = 783); a replication phase in twin pairs discordant for aggression (*N* = 378); and a validation phase in clinical cases and matched twin controls (*N* = 367). In the discovery phase, 6 biomarkers were significantly associated with childhood aggression, of which the association of O-phosphoserine (β = 0.36; *SE* = 0.09; *p* = 0.004), and gamma-L-glutamyl-L-alanine (β = 0.32; *SE* = 0.09; *p* = 0.01) remained significant after multiple testing. Although non-significant, the directions of effect were congruent between the discovery and replication analyses for six biomarkers and two neurotransmitter ratios and the concentrations of 6 amines differed between low and high aggressive twins. In the validation analyses, the top biomarkers and neurotransmitter ratios, with congruent directions of effect, showed no significant associations with childhood aggression. We find suggestive evidence for associations of childhood aggression with metabolic dysregulation of neurotransmission, oxidative stress, and energy metabolism. Although replication is required, our findings provide starting points to investigate causal and pleiotropic effects of these dysregulations on childhood aggression.

## Introduction

Biomarkers are of interest in etiological research, or as applications in clinical practice as either diagnostic or predictive instruments in personalized medicine (1). In general, a biomarker is a measurable characteristic that can serve as an indicator of the presence or absence of a trait or disorder, as an indicator of severity, or to distinguish subgroups (2). Biomarkers can be molecules, genes, or characteristics from invasively or non-invasively collected biomaterials, for example blood or urine, and may also include measures of some biological state like neuroimaging or resting heart rate (2). This paper focuses on childhood aggressive behavior and addresses the question to what extent variation in aggressive behavior is associated with biomarkers assessed in urine, which is a tissue that can be obtained non-invasively. Aggressive behavior is common in children and shows considerable individual variation, with more pathological levels of aggression thought to be at the extreme end of a continuous phenotype (3). Because of the large impact of aggression problems on children, their families, teachers, and their broader environment, there is a substantial interest in studying aggression from a wide range of disciplines, including genome, biomarker, and exposome research (4).

Aggression can be defined as a behavior that intends to cause physical or emotional harm to others (5, 6). Odintsova et al. (7) summarized all reviews of genetic studies in human aggression, including an overview of “What is considered to be aggression?” They indicated that the definitions of aggression vary considerably, ranging from broadly-defined externalizing and antisocial behaviors, including rule-breaking behavior, to narrow definitions of chronic physical aggression. The broader definitions entail a range of behaviors, which are expressed differently with age (8, 9). For example, physical aggression peaks in early childhood around 42 months (10, 11), while relational aggression increases during adolescence (12). Decreases in specific types of aggression can reflect actual cessation from aggression, while sometimes a transition is made to types of aggression which are more cognitively demanding, for example, from physical aggression into relational aggression (13). Aggression rarely occurs in isolation, and aggressive children often experience co-occurring behavioral and social problems (14, 15).

A review of the biochemical biomarker literature on aggressive behavior indicated a possible role of inflammation, neurotransmitters, lipoproteins, and several classes of hormones (16). Particularly, research has focused on the role of neurotransmitter pathways in aggressive behavior. In general, it has been hypothesized that the dopaminergic system is involved in the initiation of aggressive behavior, the serotonergic system regulates the inhibition of aggression, while the appraisal of aggression-related cues is controlled by the gamma-aminobutyric acid (GABA) system (17). Most biomarker studies of aggression have been done in adults, and much of the biochemical biomarker research is on a limited range of biomarkers (16). As a consequence, it is often unknown whether changes in selected biomarkers reflect accurate representations of their putatively associated biological pathways or systems.

Recent advances in high-throughput technologies have enabled the transition to more holistic approaches in biomarker discovery in the form of metabolomics (18). Metabolomics allow for the measurement of a large number of metabolites, which are small molecular intermediates and products of metabolism, such as amino acids, lipids, sugars, and nucleic acids (19). Metabolomics profiles represent a functional read-out of the physiological state of the human body (20, 21). With the complex and heterogeneous nature of aggression, the combination of multiple biomarkers through metabolomics, as compared with single biomarkers, may reflect its etiology more comprehensively, and provide further insight into underlying biological processes (22, 23). Metabolomics approaches may identify more informative markers, while knowledge from single biomarker studies can guide the selection of pathways most relevant to aggression (16). Two classes of compounds that are likely to be important in the study of aggression are organic acids, which play vital roles in critical metabolic pathways and neurotransmitter turnover (24), and biogenic amines. Neurotransmitters like serotonin, dopamine, norepinephrine, epinephrine, and histamine are all biogenic amines (25).

Here we present the first results from a large study on the association of childhood aggression with urinary amines, and organic acids in school-aged children (average age 9.7 years). The study is part the ACTION project [Aggression in Children: Unraveling gene-environment interplay to inform Treatment and InterventiON strategies; (4, 14)]. ACTION is a large collaborative endeavor which includes genome-wide genetic and epigenetic association studies, biomarker discovery, and epidemiological projects into the antecedents, characteristics, and consequences of childhood aggression. We describe the biomarker component of the ACTION project with a focus on metabolomics. ACTION has collected data for two metabolomics platforms, targeting amines and organic acids, as well as some other biomarkers of larger molecular weight: creatinine (indicator of renal health), neopterin (infection marker), oxidized DNA/RNA (oxidative stress marker), the neuropeptide Substance P, and C-peptide (indicator of insulin production). Participants were recruited from the Netherlands Twin Register (NTR; *N* = 1,349) and from an academic center for child and youth psychiatry in the Netherlands (Curium-LUMC, Oegstgeest; *N* = 183). We developed a standardized protocol for the large-scale collection of urine samples in children, which has been made available to the scientific community (http://www.action-euproject.eu/content/data-protocols).

The two aims of this paper were to examine whether concentrations of urinary metabolites and some larger, selected, biomarker differed between children scoring low and high on aggressive behavior and to see if we could validate the role of neurotransmitter pathways in childhood aggression. Therefore, we applied an analytical design consisting of three phases, each conducted in independent samples. First, the discovery phase assessed if aggression status was associated with urinary biomarkers levels in a sample of twins concordant for high or low aggression. Second, in the replication phase, the levels of the top 25% most strongly associated biomarkers were compared within twin pairs discordant for aggression, i.e., pairs selected in which one twin scored high and the co-twin scored low. Third, in the validation phase we assessed the top biomarkers for childhood aggression in a sample of aggressive clinical cases and low scoring twins (controls). The second aim of this paper was to examine whether we could validate the role of serotonergic, dopaminergic, and GABAergic neurotransmitter pathways in aggressive behavior for children. To do so, we used ratios of metabolites involved in neurotransmitter anabolism (synthesis) and catabolism (degradation) in the same analytical design as described above. A series of follow-up analyses was done in which case-control status was defined at the level of the individual items. We used the same analytical design, with a discovery, replication, and validation step.

## Materials and Methods

### Study Population and Procedures

#### Twin Cohort

Twins from the longitudinal Netherlands Twin Register [NTR; (26, 27)] were invited for participation in the biomarker study based on their longitudinal data on aggressive behavior at ages 3, 7, and/or 9/10 years. At, or around these ages, parents of twins received surveys that included the Dutch version of the Achenbach System of Empirically Based Assessment (ASEBA) Child Behavior Checklist (CBCL) for pre-school children (1.5–5 years) or school-aged children [6–18 years; (28)]. Maternal data were always collected, paternal ratings are missing for some birth cohorts due to financial constraints. At ages 7 and 9/10, teachers of twins also received surveys that included the Dutch version of the ASEBA Teacher Rating Form [TRF; (28)] after parents consented to approach the teachers and provided contact information. Twin pairs were invited for participation in the biomarker study based on concordance or discordance for aggressive behavior rated by either the mother (93%) or teacher(s; 7%) on the Aggressive Behavior subscale of the CBCL/TRF, with an intentional oversampling of monozygotic (MZ) pairs. The design included twins from high-high and low-low scoring concordant pairs, and twins from discordant high-low pairs (81% MZ pairs). NTR defined age- and sex-specific Aggressive Behavior T-scores by multiplying a z-score by 10 and adding 50. High-scoring children had T-scores ≥ 65. Low-scoring children had sum scores lower than five. We selected high-high, low-low, and high-low pairs based on these criteria and additionally matched low-low pairs to the other pairs based on postal code. In the last phase of recruitment, an age-specific sum score defined high-scoring children based on mother ratings as: age 3 ≥ 13, age 7 ≥ 5, and age 10 ≥ 4.

Prior to biological sample collection in the twin cohort, a feasibility study established achievability of urine collection and storage in the home context. Parents collected first-morning urine samples (see Supplementary Text 1 for description of buccal cell collection). Urine samples were stored at home and transported by researchers to the lab at −18 degrees Celsius. In the lab, urine samples were stored at −80°C until further processing. All parents provided written informed consent for their children's participation. At the time of sample collection, they answered a set of questions about the precise dates and times of urine collection, their children's general health, and current medication use. Parents also completed the CBCL, of which the Aggressive Behavior subscale was used to measure the twins' aggressive behavior at the time of urine collection.

From December 2014 to May 2017, 3,304 twins were invited with 1,367 twins (41.4%) agreeing to take part. The invited group comes from the larger Netherlands Twin Register. Heritability estimates of aggression were calculated from CBCL Aggression scores of the entire twin sample from which the twins who were invited into the biomarker study were drawn. The ACTION-biomarker project included 1,362 twins with first-morning urine (Supplementary Table 1). Twins were excluded if the collected urine was not the first-morning urine (e.g., parent-reported time of urine collection was after 12:00 in the afternoon; *N* = 13) or if the urine sample was too small to analyze both metabolomics platforms and all biomarkers (*N* = 2). This resulted in a total of 1,347 urine samples (673 complete twin pairs) in which analyses were performed. Study approval was obtained from the Central Ethics Committee on Research Involving Human Subjects of the VU University Medical Center, Amsterdam (NTR 25th of May 2007 and ACTION 2014.252), an Institutional Review Board certified by the U.S. Office of Human Research Protections (IRB number IRB00002991 under Federal-wide Assurance- FWA00017598; IRB/institute codes).

#### Clinical Cohort

Six- to 13-years-old children were recruited who were referred to an academic center for child and youth psychiatry in the Netherlands (Curium-LUMC) between February 2016 and June 2018. This center provides inpatient and outpatient treatment programs and treats children with severe and complex mental health problems who are in need of intensive care. As part of a standardized clinical assessment, parents completed the Dutch version of the CBCL (28), of which the Aggressive Behavior subscale was used as an index of aggression. These data were made available to the authors for the purpose of the present study. Specifically, parents were approached in the context of an ongoing biobank protocol approved by the ethics board of Leiden University Medical Center. For children for whom parents agreed to participate, biomaterials (buccal cells and urine) and physical measures (height, weight, resting heart rate) were also collected. Collection of biomaterials was identical to the twin sample's procedure. In total, 809 parents and children were invited to participate in the study, of which 189 (23.4%) agreed to participate (including eight sibling pairs and sibling trio). Several children refused to participate during urine collection (*N* = 3) or donated urine in the afternoon (*N* = 2). One child was excluded as this child and its co-twin were also included as part of the twin cohort. This resulted in a total of 183 clinical cases with urine samples available. Information on psychiatric disorders in the clinical sample is available in Supplementary Table 2.

For the 183 clinic-referred children who donated morning urine (mean age = 10.2 years, *SD* age = 1.8; 25.7% female), 180 children had CBCL parent reports available and 164 children also had TRF teacher reports. ASEBA questionnaires were completed a maximum of 6 months before or after urine collection. All clinic-referred children were considered aggressive cases in our design, which was confirmed by the ASEBA sex-specific norm scores. Specifically, the clinical sample displayed subclinical levels of parent-rated CBCL aggression with average T-scores of *M* = 66.08 (*SD* = 11.13), with T ≥ 65 conferring to subclinical levels of aggression, and T ≥ 70 to clinical levels of aggression. Teacher-reported aggression was substantially elevated in the clinical sample with an average T-score of *M* = 60.45 (*SD* = 8.19), with a score of *T* = 60 referring to one standard deviation elevation above the sample mean.

### Biomarker Measurement

#### Biomarker Quantification

##### Dipstick

A dipstick (Siemens, Marburg, Germany) was used to screen for infections in urine and to measure leukocytes, nitrite, proteins, glucose, and blood presence in the urine. The dipstick was applied to the first thaw of the urine samples either by dipping in the residual urine volume after aliquoting or by dropping urine on the dipstick. No children had to be excluded.

##### Density

Density of urine was measured using the Atago® refractometer PAL-10S BLT/A+W (Atago, Tokyo, Japan). The refractive index is a ratio of the velocity of light in air to the velocity of light in solution, which is directly proportional to the number of dissolved solids in urine.

##### Creatinine

Creatinine was measured using a colorimetric assay kit according to manufacturer's instructions (Cayman, Ann Harbor, MI, USA). Creatinine values are reported in μmol/L.

##### Neopterin

Neopterin is a peptide which responds to damage and infection, especially to tissue damage and viral infection. Neopterin was measured using a competitive ELISA according to manufacturer's instructions (IBL International GmbH, Munich, Germany) Neopterin levels are reported in nmol/L.

##### Oxidized DNA/RNA

DNA and RNA are damaged by oxidation, with guanine as most prone to oxidation. Using a competitive ELISA (Cayman, Ann Harbor, MI, USA), different oxidized guanine species were measured in urine including 8-hydroxyguanosine, 8-hydroxy-2'-deoxyguanosine, and 8-hydroxyguanine. We used these oxidized guanine species as marker for oxidized DNA and RNA. Oxidized DNA/RNA levels are reported in pg/ml.

##### C-peptide

Insulin is synthesized in the pancreatic beta cells as proinsulin. Proinsulin is cleaved enzymatically, releasing insulin and its byproduct C-peptide. C-peptide was measured using an ELISA according to manufacturer's instructions (IBL International GmbH, Munich, Germany) and was used as a marker of insulin in urine. C-peptide levels are reported in ng/ml.

##### Substance P

The peptide neurotransmitter substance P was measured in urine using competitive ELISA according to manufacturer's instructions (Cayman, Ann Harbor, MI, USA). Substance *P* levels are reported in pg/ml.

#### Metabolite Quantification

##### LC-MS amines platform

The amine metabolites were measured using ultra-performance liquid chromatography tandem mass spectrometry (UPLC-MS/MS) employing an Accq-Tag derivatization strategy adapted from the protocol supplied by Waters. Sample preparation consisted of protein precipitation by the addition of methanol to 5 μL of urine spiked with internal standards. The centrifuged supernatant was then evaporated using a speedvac prior to reconstitution in borate buffer (pH 8.5) with AQC reagent. Chromatic separation was done on an Accq-Tag Ultra column (Waters Chromatography B.V., Etten – Leur, The Netherlands) using a UPLC Agilent Infinity II (1290 Multisampler, 1290 Multicolumn Thermostat and 1290 High Speed Pump; Agilent Technologies, Waldbronn, Germany) coupled to an AB SCIEX quadrupole-ion trap (QTRAP; AB Sciex, Massachusetts, USA). Analytes were detected in the positive ion mode and monitored in Multiple Reaction Monitoring (MRM) using nominal mass resolution. The amine method has been described in detail elsewhere (29). Metabolites are reported as ‘relative response ratios' (target area/area of internal standard) after quality control (QC) correction.

##### GC-MS organic acids platform

The organic acid metabolites were measured using gas chromatography mass spectrometry (GC-MS). Sample preparation of 50 μL of urine spiked with internal standards consisted of liquid-liquid extraction with ethyl acetate to extract the organic acids and remove urea present in the urine. After collecting the organic phase, the samples were evaporated to dryness using a speedvac. Then, two-step derivatization procedures were performed on-line: oximation using methoxyamine hydrochloride (MeOX, 15 mg/mL in pyridine) as first reaction and silylation using N-Methyl-N-(trimethylsilyl)- trifluoroacetamide (MSTFA) as second reaction. Chromatic separation using helium as carrier gas (1,7 mL/min) was performed on a 30 × 0.25 m ID column with a film thickness of 25 m (HP-5MS UI). The mass spectrometer (Agilent Technologies, Waldbronn, Germany) with a single quadrupole using electron impact ionization (70 eV) was operated in SCAN mode (mass range 50–500). Metabolites are reported as “relative response ratios” (target area/area of internal standard) after QC corrections. The acceptance criteria for metabolite reporting was a relative standard deviation (RSD) of the QCs (RSDqC) of <15% and background signal <20%, metabolites with RSDqc values of 15–30% should be interpreted with caution.

##### Metabolomics measurement protocol

In order to minimize the analytical error in the data, a number of measures were taken. A QC sample was created by pooling aliquots from all urine samples. Randomization of the subjects was done in such a manner that low and high aggression subjects, and therefore twin and clinical samples were randomly distributed across batches. Twin pairs were included in the same batch. Samples were run in 20 batches which included a calibration line, QC samples, sample replicates and blanks. QC samples were analyzed every 10 samples, and used to assess data quality and to correct for instrument response. Blank samples were used to determine if there was any interference from background signal. In-house developed algorithms were applied using the pooled QC samples to compensate for shifts in the sensitivity of the mass spectrometer over the batches. The performance and reproducibility of individual metabolites were evaluated with the RSDqc. The acceptance criteria for metabolite reporting was RSDqc <15% and background signal <20%, metabolites with RSDqc of 15–30% should be interpreted with caution.

### Data Pre-processing for Analysis

Preprocessing of the metabolomics data was done for each platform. To avoid the exclusion of potentially relevant metabolites and to avoid including metabolites with very poor RSDqc values, metabolites with a RSDqc value of >20% were removed (RSDqc values are given in Supplementary Table 3). Metabolite measurements that fell below the limit of detection/quantification were imputed with half of the value of this limit, or when this limit was unknown with half of the lowest observed level for this metabolite (the number of imputed values per metabolite have been included in Supplementary Table 3). Urine volume fluctuates among individuals and throughout the day; therefore, correction for dilution in urinary metabolite concentrations is essential. It is common practice to normalize to urinary creatinine output to correct for dilution differences (30). However, creatinine was associated with childhood aggression (unpublished pilot study), therefore, normalization to creatinine levels would bias our results. Instead we applied an adjusted variant of density normalization. The density reflects the dilution of the urine sample and thus can be used to account for hydration state of the subject. In a healthy representative population, one can account for hydration state by dividing the metabolite concentrations by (*d*_*i*_–*d*_*w*_), where *d*_*i*_ is the density of sample *i and d*_*w*_ = 1 the density of pure water. In this study, we took the data from the control group to construct the linear models that predict the concentration of each metabolite from the density measure. The density effect size β_*m*_ for each metabolite *m* is then used as a scaling factor in the density normalization for the entire population as follows:

$$\begin{array}{l} {\left[m_{i}\right]}^{\prime }=\left[m_{i}\right]/\left({\beta_{m}}^{*}\left(d_{i}-d_{w}\right)\right), \end{array}$$

where [*m*_*i*_] denotes the measured concentration of metabolite m in sample *i* and [*m*_*i*_]' the corrected concentration. For convenience, densities and concentrations are expressed as a percentage of their median. The regression parameters are all listed in Supplementary Table 3. In generating the models, we imputed data points that deviated more than 2.5 *SDs* from the mean by the mean metabolite or biomarker concentration. After normalization we verified if the effect of density on [*m*_*i*_]' disappeared as one would expect. This was indeed the case by considering data points within 3 *SDs* from the mean for each metabolite, c.f. Supplementary Table 3. Finally, the metabolites and biomarkers were transformed by inverse normal rank transformation (31, 32).

To get an indication of the metabolic functioning of serotonergic, dopaminergic, and GABAergic neurotransmitter pathways, ratios were calculated between metabolites which have been associated with these pathways. Specifically, we targeted serotonergic, dopaminergic, and GABAergic anabolism (synthesis) and catabolism (degradation). Serotonergic anabolism was represented by the ratios of L-tryptophan to 5-hydroxy-L-tryptophan (5HTP) and 5HTP to serotonin. Dopaminergic anabolism was assessed with the ratio of L-phenylalanine to L-tyrosine, while the ratio of 3-methoxytyramine (3MT) to homovanillic acid (HVA) represented dopamine catabolism. The ratios of L-glutamine to L-glutamic acid and L-glutamic acid to GABA represented GABA synthesis and GABA to succinic acid GABA degradation.

### Statistical Analyses

Because twins were selected for the biomarker study on the basis of prior longitudinal data, it was important to assess whether these group differences in aggression were still present at the time of urine collection. Generalized estimation equation (GEE) models tested whether twins selected for high or low aggression and clinical cases and twin controls differed in aggressive behavior at the time of urine collection (see Main analyses for details on GEE analyses). Similarly, a paired sample *t*-test was used to assess differences within twin pairs discordant for aggression (i.e., high co-twin vs. low co-twin). All analyses were carried out in the R programming language [version 3.6.0; (33)]. For the entire NTR group (1,502 MZ twins and 2,298 DZ twins), from which the ACTION-biomarker subsample was drawn, we analyzed the CBCL aggression scores with genetic structural equation modeling (34, 35) to obtain estimates of heritability, influences of shared (common), and unshared (unique) environmental factors.

#### Analytical Design

We employed a three-step analytical strategy, with independent samples included in each step: (1) discovery in between-family analyses; (2) replication in within-family analyses; and (3) validation in clinically referred aggression cases and twin controls. In the discovery phase we explored the differences between high and low concordant twin pairs in biomarkers levels and neurotransmitter ratios in between-family analyses with GEE models. The within-family replication analyses were performed for the top 25% most strongly associated biomarkers or ratios from the discovery phase. In the within-family analyses we compared the biomarker levels and neurotransmitter ratios of the low and high scoring twin of discordant twin pairs. Finally, for the biomarkers or ratios that differed consistently for aggression status in the discovery and replication phase we performed validation analyses to compare the levels of these biomarkers or the neurotransmitter ratios between clinical cases and controls.

Sensitivity analyses were performed to assess the impact of confounders (preexisting chronic conditions, medication use, and vitamin supplementation) on the results. After removal of individuals scoring positive on these potential confounders, we repeated the within-family analyses, for the biomarkers and ratios included in the replication phase of the analytical strategy.

#### Main Analyses

The between-family discovery analyses included the twins scoring high or low on aggression. To investigate the first aim of the study, the relation of amines, organic acids, and biomarkers with childhood aggression, GEE analyses were performed to model the relationship between biomarkers (outcomes) and aggression status (predictors), with sex and age at urine collection as covariates. The second aim of this study, to investigate the contribution of neurotransmitter pathways (i.e., serotonergic, dopaminergic, and GABAergic) to aggression, was explored through identical GEE models, except with neurotransmitter ratios as outcomes. Aggression case-control status was the predictor in all analyses. GEE uses a sandwich or robust variance estimator that adjusts the standard errors to correct for clustering in the data (36). In our analyses the clustering in the data is due to relatedness of participants (i.e., twins within families), to correct for this we used the “exchangeable” correlation structure option in GEE. To correct for multiple testing (p.adjust function in R) we used the False Discovery Rate [FDR; (37)] of 5% for 89 (biomarkers) or 7 (ratios) tests, the significance threshold was set at *p* ≤ 0.05.

The within-family replication analyses was done in twin pairs that were discordant for aggression status (high-low) and tested the top 25% most strongly associated biomarkers or ratios from the between-family analyses. Biomarker concentrations or ratios were corrected for the effects of sex and age at urine collection by regressing out their effects. We then employed paired *t*-tests to analyze the residuals of the regression analysis. The FDR of 5% for 23 (biomarkers) or 3 (ratios) tests was used to correct for multiple testing, with the significance threshold at *p* ≤ 0.05.

The top five most strongly associated biomarkers and top ratio were included in the validation analyses; these were required to have the same direction of effect in both the discovery and validation analyses. To assess if levels of the biomarkers and ratio selected by the discovery and validation analyses can differentiate between low and high aggressive children, we performed replication analyses in clinical cases and twin controls (92 twin pairs not previously included in the discovery between-family analyses). As for the discovery analysis, we performed GEE analyses to model the relationship of the biomarkers and ratio with aggression status. Sex and age at urine collection were included as covariates and we used to “exchangeable” correlation structure to correct for relatedness in our sample and obtain robust standard errors. For the biomarkers we used the FDR of 5% for 5 tests to account for multiple testing, *p* ≤ 0.05 was considered significant.

#### Sensitivity Analyses

Sensitivity analyses were done in the discordant monozygotic twin pairs, and comprised of the biomarkers and the neurotransmitter ratio included in the validation phase. These analyses only included data from twins without a preexisting chronic condition (*N* = 24 excluded), who were medication (*N* = 48 excluded) or vitamin supplement (*N* = 67 excluded) naive (see Supplementary Text 2 for more information). After exclusions, we performed paired *t*-tests to re-evaluate the differences in biomarker levels and the neurotransmitter ratio between the aggressive and non-aggressive twins. The FDR of 5% for 15 (biomarkers) or 3 (ratios) tests was used to correct for multiple testing, with the significance threshold at *p* ≤ 0.05.

Finally, we carried out sensitivity analyses on item level data (see Supplementary Table 4). These sensitivity analyses entailed association analyses of each metabolite, other biomarker of neurotransmitter ratio with each item from the CBCL Aggressive Behavior subscale (see Supplementary Text 3).

## Results

### Participant and Aggression Description

The present study contains data from 1,530 children, including twins and clinical cases, aged 9.7 years on average (range 5.6 to 13.4 years; *SD* = 1.8) of which 693 (45.3%) were females. In total, we included 794 (51.9%) children scoring low on aggression and 736 (48.1%) children with a high aggression score (Table 1). Twin pairs were invited for participation based on longitudinal data on childhood aggressive behavior (Supplementary Table 5 and Supplementary Text 4). We compared the CBCL aggression scores, obtained at time of urine collection, to assess whether differences in aggression between the high and low scoring twins were still present at the time of urine collection. At the time of urine collection, twins selected for high aggression indeed had significantly higher CBCL aggression scores as compared to twins selected for low aggression (β = 5.09; *SE* = 0.50; *p* = 1.83 × 10^−24^). Similarly, when comparing the discordant twin pairs, the high aggressive twins (*M* = 6.2, *SD* = 5.8) had significantly higher aggression scores at the time of urine collection than their low aggressive co-twins (*M* = 4.4, *SD* = 4.4; *t*(185) = 5.73, *p* = 4.08 × 10^−08^). Finally, the clinical cases and low aggressive twin controls, differed greatly in their levels of aggression (β = 10.19; *SE* = 0.74; *p* = 8.25 × 10^−43^). The heritability of the CBCL aggression scores as analyzed in our project was 0.63 (90% CI: 0.53–0.74). The proportion of variation explained by common environment shared by twins growing up in the same family was 0.14 (90% CI: 0.03–0.24) and the proportion of variation explained by unique environment was 0.23 (90% CI: 0.21–0.25).

**Table 1:** Participant characteristics of the twins (*N* = 1,347) and clinical cases (*N* = 183).

	**Twins**				**Clinical cases**
	**Concordant low**	**Discordant**		**Concordant high**	
	***n* = 605**	**Low (*n* = 189)**	**High (*n* = 189)**	***n* = 364**	***n* = 183**
N complete twin pairs	302	189		182	-
Mean (SD) age sample collection	9.4 (1.9)	10.1 (1.7)		9.5 (1.8)	10.2 (1.8)
Range age sample collection	5.6–12.6	6.1–12.7		5.8–12.9	6.3–13.4
N (%) MZ twins	469 (77.5%)	306 (81.0%)		330 (90.7%)	
N (%) females	323 (53.4%)	85 (45.0%)	79 (41.8%)	159 (43.7%)	47 (25.7%)
CBCL mother (SD) aggression score	2.7 (3.8)	4.4 (4.4)	6.2 (5.8)	7.6 (6.0)	13.0 (7.6)
**Current psychotropic medication use**					
Stimulants	10 (1.7%)	7 (3.7%)	13 (6.9%)	25 (7.0%)	46 (24.6%)
Analgesics	1 (0.2%)	1 (0.5%)	3 (1.6%)	1 (0.3%)	0 (0.0%)
Antipsychotics	1 (0.2%)	0 (0.0%)	1 (0.5%)	3 (0.8%)	36 (19.7%)
Hypnotics/sedatives	7 (1.2%)	1 (0.5%)	2 (1.1%)	6 (1.7%)	6 (3.3%)

*CBCL, Child Behavior Checklist; MZ, monozygotic. The clinical cases CBCL scores include either mother of father-report (90% mother report)*.

### Association of Urinary Metabolites and Other Biomarkers With Childhood Aggression

#### Discovery Analyses

To determine the association of urinary amine, organic acid, and biomarker levels with childhood aggression, we first performed discovery analyses using a between-family design. The discovery analyses were conducted using 421 low scoring and 364 high scoring twins (average age = 9.4; *SD* = 1.8) and included 48.8% females and 84% MZ twins (Table 1). The discovery analyses showed significant associations for 4 amines and two other biomarkers with childhood aggression. We observed positive associations of childhood aggression with creatinine (β = 0.24; *SE* = 0.08; *p* = 0.003; FDR *p* = 0.08), oxidized DNA/RNA (β = 0.19; *SE* = 0.09; *p* = 0.03; FDR *p* = 0.54), and L-methionine sulfoxide (β = 0.18; *SE* = 0.09; *p* = 0.04; FDR *p* = 0.57) and negative associations with gamma-glutamylglutamine (β = −0.25; *SE* = 0.09; *p* = 0.004; FDR *p* = 0.09; Supplementary Table 7). After correction for multiple testing, the positive associations of O-phosphoserine (β = 0.36; *SE* = 0.09; FDR *p* = 0.004), and gamma-L-glutamyl-L-alanine (β = 0.32; *SE* = 0.09; FDR *p* = 0.01) remained significant (Supplementary Table 6).

#### Replication Analyses

**Figure 1:**
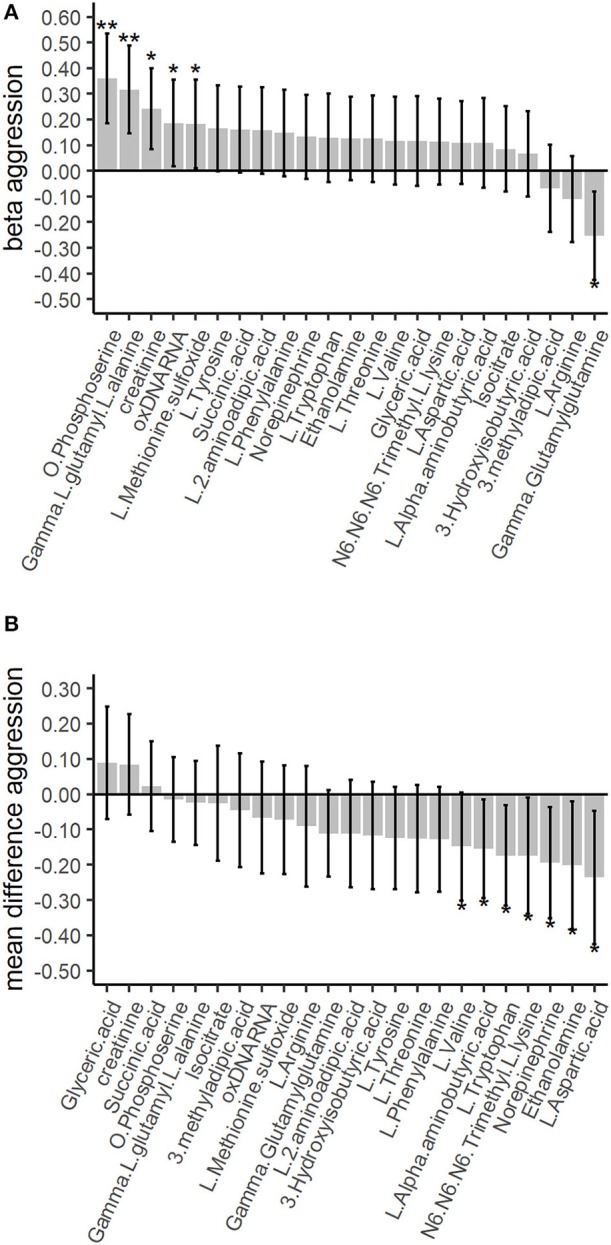
Association of the top 25% amines, organic acids and other biomarkers with childhood aggression in the discovery and validation phases. The between-family analyses in the discovery phase are based on gee models for the 783 twins scoring low or high aggression. The within-family analyses in the validation phase are based on paired *t*-tests among the 189 twin pairs discordant for aggression. The whiskers denote the 95% confidence intervals for the GEE betas or the mean differences. Single asterisk represents a significant finding before correction for multiple testing at *p* ≤ 0.05, double asterisks represent a significant finding after correction for multiple testing. **(A)** The top 25% between-family results for the amines, organic acids and other biomarkers in the discovery phase. Correction for multiple testing was done with the False Discovery Rate (FDR) of 5% for 89 tests. **(B)** The top 25% within-family results for the amines, organic acids and other biomarkers. Correction for multiple testing was done with the FDR of 5% for 23 tests.

The top 25% most strongly associated amines (16), organic acids (5), and biomarkers (2) from the discovery analysis were examined in within-family analyses, conducted in 189 twin pairs discordant for childhood aggression status (Table 1). There was no replication of associations with childhood aggression of the discovery phase, where 2 amines were significantly associated after correction for multiple testing (Supplementary Table 7). As compared to their low aggression co-twin, twins with high aggression had significantly lower concentrations of L-aspartic acid (mean difference = −0.24; *t*(188) = −2.46; *p* = 0.01), norepinephrine (mean difference = −0.19; *t*(188) = −2.44; *p* = 0.02), L-tryptophan (mean difference = −0.17; *t*(188) = −2.40; *p* = 0.02), ethanolamine (mean difference = −0.20; *t*(188) = −2.20; *p* = 0.03), L-alpha-aminobutyric acid (mean difference = −0.16; *t*(188) = −2.20; *p* = 0.03), and N6-N6-N6-trimethyl-L-lysine (mean difference = −0.17; *t*(188) = −2.09; *p* = 0.04; Supplementary Table 7). However, none of these associations survived multiple testing (Supplementary Table 7). Overall, we observed congruent directions of effect in the discovery and validation analyses for 6 out of 23 (26.1%) top 25% amines, organic acids and biomarkers (Figure 1).

#### Validation Analyses

To assess if biomarkers selected in the discovery and replication analyses could differentiate between low and high aggressive children from an independent sample, we analyzed data from 183 clinical cases and 184 controls (92 twin pairs concordant low for childhood aggression). This validation sample included children with an average age of 9.8 years (*SD* = 1.9), 39.8% females and 38.2% MZ twins. The analyses included the top 5 biomarkers with congruent direction of effect in the discovery and validation analyses: gamma-glutamylglutamine, L-arginine, glyceric acid, creatinine, and succinic acid. None of the biomarkers were significantly associated with childhood aggression in the validation analyses (Table 2 and Supplementary Table 8). We observed the same direction of effect in the validation analysis for 3 (60%) biomarkers (Table 2).

**Table 2:** Results of the top five associated biomarkers as included in the replication analysis.

	**Platform**	**Amines**	**Amines**	**Other**	**Organic acids**	**Organic acids**
	**Metabolite**	**Gamma-glutamylglutamine**	**L-arginine**	**Creatinine**	**Glyceric acid**	**Succinic acid**
**Discovery analysis**	N	783	783	785	783	783
	B	−0.25	−0.11	0.24	0.12	0.16
	SE	0.09	0.08	0.08	0.09	0.09
	*p*-value	**0.004**	0.193	**0.003**	0.193	0.060
	FDR *p*-value	0.09	0.79	0.08	0.79	0.66
**Replication analysis**	Mean difference	−0.11	−0.09	0.08	0.09	0.02
	df	188	188	188	188	188
	T	−1.80	−1.05	1.17	1.09	0.34
	*p*-value	0.07	0.29	0.24	0.28	0.73
	FDR *p*-value	0.21	0.42	0.40	0.42	0.79
**Validation analysis**	N	367	367	367	367	367
	B	−0.17	0.12	0.16	0.09	−0.23
	SE	0.14	0.11	0.12	0.12	0.13
	*p*-value	0.25	0.27	0.16	0.42	0.07
	FDR *p*-value	0.34	0.34	0.34	0.42	0.34

*This table includes the results from the between-family discovery, the within-family validation and replication analyses for all 5 biomarkers. Discovery analyses were performed with GEE for 783 twins with low or high aggression. The p-values in the discovery analysis have been adjusted for multiple testing using the FDR of 5% for 89 tests. Validation analyses were performed with paired t-tests for 189 twin pairs discordant (high-low) on aggression status. The p-values in the validation analysis have been adjusted for multiple testing using the FDR 5% for 23 tests. Replication analyses were performed with GEE for 183 clinical cases and 184 twin controls. The p-values in the replication analysis have been adjusted for multiple testing using the FDR of 5% for 5 tests. All p ≤ 0.05 have been given in bold. Full model information of the discovery, validation and replication analyses have been included in Supplementary Tables 6–8, respectively*.

#### Sensitivity Analyses

For the five biomarkers included in the validation analyses we performed sensitivity analyses to assess if the mean difference between high and low aggressive children changed after excluding twins with potentially confounding characteristics (preexisting chronic condition, currently on medication, or on vitamin supplements). As compared to the within-family analyses (Table 2), we observed no differences after exclusions for preexisting chronic disorder, medication or vitamin use for any of the biomarkers (Supplementary Table 9).

Item-based analyses found no significantly associated metabolites or other biomarkers after correction for multiple testing. Replication and validation analyses also found no significant metabolites or other biomarkers per item after correction for multiple testing (Supplementary Tables 13–16). The complete results have been included in Supplementary Text 3.

### Association of Urinary Neurotransmitter Pathways in Childhood Aggression

#### Discovery Analyses

To elucidate the role of serotonergic, dopaminergic, and GABAergic neurotransmitter pathways in childhood aggression we analyzed neurotransmitter ratios representing anabolism (synthesis) and catabolism (degradation) of the key neurotransmitters in these pathways. The discovery analyses using a between-family design to assess the association of urinary neurotransmitter ratios with childhood aggression found no neurotransmitter ratios involved in the anabolism or catabolism of serotonin, dopamine or GABA significantly associated with childhood aggression (Supplementary Table 10).

#### Replication Analyses

**Figure 2:**
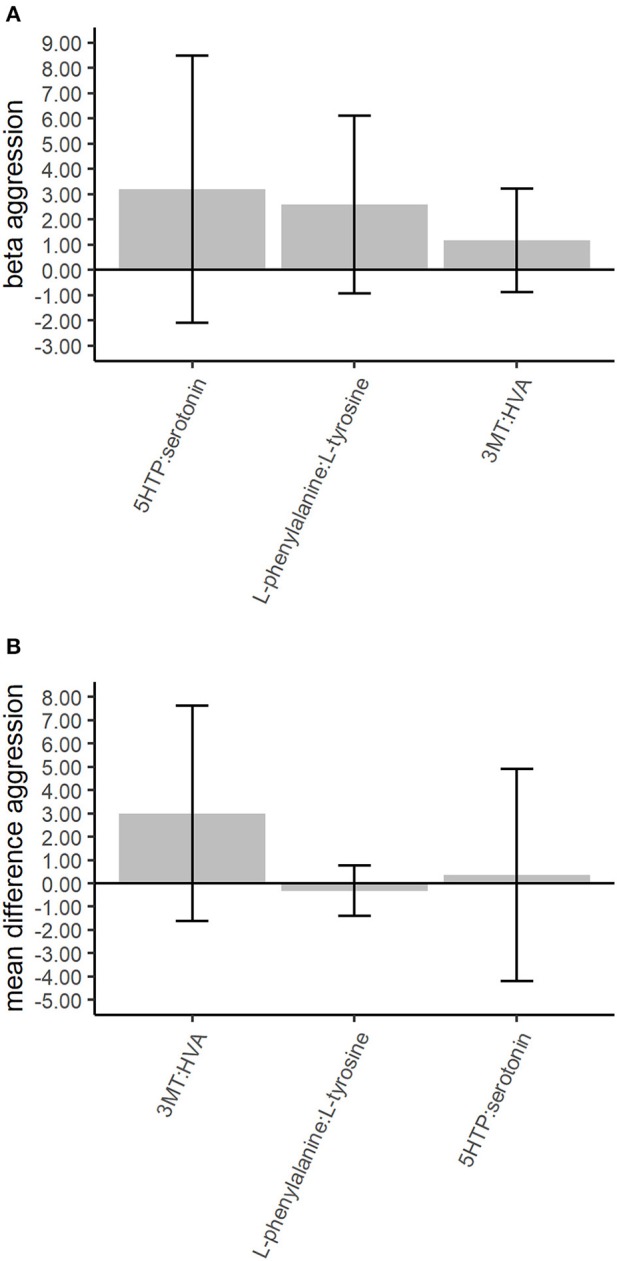
Association of the top 25% neurotransmitter ratios with childhood aggression in the discovery and validation phases. The between-family analyses in the discovery phase are based on gee models for the 783 twins scoring low or high aggression. The within-family analyses in the validation phase are based on paired *t*-tests among the 189 twin pairs discordant for aggression. The whiskers denote the 95% confidence intervals for the GEE betas or the mean differences. The neurotransmitter ratios denote the following: 3MT, 3-methoxytyramine; 5HTP, 5-hydroxy-L-tryptophan; HVA, homovanillic acid. **(A)** The top 25% between-family results for the neurotransmitter ratios in the discovery phase. Correction for multiple testing was done with the False Discovery Rate (FDR) of 5% for 7 tests. **(B)** The top 25% within-family results for the neurotransmitter. Correction for multiple testing was done with the FDR of 5% for 3 tests.

Replication in the top 25% most strongly associated neurotransmitter ratios (3) from the discovery analysis were done in within-family analyses. The 3 top 25% neurotransmitter ratios included the dopamine ratios 3MT:HVA and L-phenylalanine:L-tyrosine and the serotonergic ratio 5HTP:serotonin. None of the neurotransmitter ratios showed significant differences between high and low aggressive twins (Supplementary Table 11). We observed congruent directions of effect in the discovery and replication analyses for 2 of the 3 (66.6%) top 25% neurotransmitter ratios (Figure 2).

#### Validation Analyses

To assess if neurotransmitter ratios selected in the discovery and replication analyses could differentiate between low and high aggressive children we analyzed data from 183 clinical cases and 184 twin controls. The top neurotransmitter ratio with the same direction of effect in the discovery and replication analyses was 3MT:HVA. The catabolic dopaminergic ratio 3MT:HVA was not significantly associated with childhood aggression in a sample of clinical cases and twin controls (*B* = 2.12; *SE* = 1.57; *p* = 0.18).

#### Sensitivity Analyses

We performed sensitivity analyses to assess if the mean difference in the top neurotransmitter ratio between high and low aggressive children changed after excluding participants with potentially confounding characteristics. Excluding twins with a preexisting chronic condition or who were on medication or vitamin supplements, did not result in significant differences between aggressive and non-aggressive twins for the 3MT:HVA ratio (Supplementary Table 12).

After correction for multiple testing none of the neurotransmitter ratios were significantly associated to any of the Aggressive Behavior items in the discovery analyses. Similarly, replication and validation analyses also found that associations of Aggressive Behavior items with neurotransmitter ratios did not survive multiple testing (Supplementary Tables 17–20). Supplementary Text 3 contains a complete description of the results.

## Discussion

Discovery of biomarkers that would aid in the diagnostics and treatment of childhood aggression could be of great benefit. To illustrate, poorer adult outcomes have been reported for later diagnosis, and thus treatment, of aggression (38). Here, we describe the first urinary metabolomics study for childhood aggression, conducted in a sample of 1,347 twins selected for high or low aggression and a sample of 183 clinically- referred children with high aggression. Our first aim was to identify metabotypes for childhood aggression based on a total of 89 amines, organic acids, and other biomarkers of larger molecular weight. The second aim was to validate the role of serotonergic, dopaminergic, and GABAergic neurotransmitter pathways in childhood aggression. Thus, we compared seven ratios of metabolites reflecting neurotransmitter anabolism (synthesis) and catabolism (degradation) between aggressive and non-aggressive children.

Out of the 89 tested amines, organic acids, and other biomarkers of larger molecular weight, we observed significant associations for 4 amines (O-pshosposerine, gamma-L-glutamyl-L-alanine, gamma-glutamylglutamine, and methionine-sulfoxide) and 2 biomarkers (creatinine and oxidized DNA/RNA) in the discovery stage before correction for multiple testing. After correction for multiple testing, only O-phosphoserine, and gamma-L-glutamyl-L-alanine remained significantly associated. None of the organic acids or neurotransmitter ratios were significantly associated with childhood aggression. The replication phase included the top 25% most strongly associated amines, organic acids, other biomarkers, and neurotransmitter ratios from the discovery phase. The replication analyses revealed significant differences between low and high aggressive twins for the levels of 6 amines (L-aspartic acid, norepinephrine, L-tryptophan, ethanolamine, L-alpha-aminobutyric acid, and N6-N6-N6-trimethyl-L-lysine). These 6 amines were among the top 25% most strongly associated traits in the discovery phase, but did not reach a significance of FDR 5%. Consequently, validation was performed on the top biomarkers (gamma-glutamylglutamine, L-arginine, glyceric acid, creatinine, and succinic acid) and neurotransmitter ratio (3MT:HVA), which had congruent directions of effect in both the discovery and replication samples. The validation analyses were conducted in an independent sample of aggressive clinical cases and non-aggressive twin controls and did not show any significant differences between groups. We compared these results to those obtained when applying sample median normalization of the metabolomics measurements and found results to be highly similar (for correlations between the beta's obtained by both normalizations: Pearson's *r* correlation 0.87, *p* = 1.89 × 10^−27^).

To assess if the heterogeneous nature of aggression prevented us from finding robust biomarkers or neurotransmitters associated with childhood aggression, all biomarkers and neurotransmitter ratios were reanalyzed for their association with endorsement of individual aggressive behavior questionnaire items. While we found some evidence for biomarkers and neurotransmitter ratios being differentially associated with distinct aggressive behaviors, like threatens or argues, none of the associations survived multiple testing.

Based on our findings for overall aggression and on the current state of the art in the field of human studies on the aetiology of aggression with respect to biomarkers, including genetic factors, hormones, and metabolites, below, we address three biochemical pathways and discuss their roles in aggression.

### Serotonergic, Dopaminergic, and GABAergic Pathways and Childhood Aggression

It has been suggested that serotonergic, dopaminergic, and GABAergic pathways play a role in aggression (17). The role of these neurotransmission systems in aggression in humans is largely based on candidate gene studies. Candidate gene studies have mainly focused on the monoamine oxidase A gene *MAOA*, the catecholamine o-methyltransferase gene *COMT* and transporter and receptor genes for dopamine and serotonin, including *5HTTP, DRD2, DRD4*, and *DRD5* (3, 7). However, results from candidate gene studies replicated poorly, and well-powered genome-wide association studies are required to determine the value of these candidate genes for aggressive behavior (7). Nevertheless, neurotransmission pathways remain interesting candidates for biomarker discovery. Therefore, we compared ratios of urinary metabolites representing anabolism (synthesis) and catabolism (degradation) of serotonin, dopamine, and GABA, between aggressive and non-aggressive children. We found no significant associations of urinary neurotransmitter ratios and childhood aggression in the discovery, replication, or validation phase. However, several of the metabolites included in our top 25% most strongly associated biomarkers indicate that dysregulation of serotonergic, dopaminergic, and GABAergic neurotransmitter pathways can be involved in childhood aggression.

In the between-family discovery analysis we observed higher, non-significant, levels of L-tryptophan in children with high aggression, though the replication analysis revealed significantly lower L-tryptophan levels in children with high aggression. A previous study reported lower serum levels of tryptophan in aggressive inmates and increases in the ratio of tryptophan to serotonin (39). Similarly, lower plasma L-tryptophan levels have been observed in patients with major depressive disorder (MDD) as compared to controls (40). In the discovery analyses we observed significantly higher levels of O-phosphoserine, an ester of serine and phosphoric acid. High levels of phosphoserine indicate dysregulation of serotonin and dopamine metabolism pathways as it expresses a lack of pyridoxal-5-phosphate (41). Due to low pyridoxal-5-phosphate levels L-tryptophan cannot be converted to serotonin, nor can the conversion of L-tyrosine to dopamine occur (41). While none of the dopamine metabolites have been included in our top 25% most strongly associated biomarkers, norepinephrine, which is synthesized through catabolism of dopamine, was included in this top 25%. The role of norepinephrine in depression and anxiety disorders is well-established (42), for example, increased plasma norepinephrine levels were observed in new mothers suffering from postpartum depression as compared to control new mothers (43). In children, plasma norepinephrine levels were correlated with inefficient conditioned pain modulation response (44). Furthermore, norepinephrine is increased by S-Adenosyl-Methionine (SAMe), which is the primary methyl group donor for several metabolic compounds and cysteine (DNA) methylation (45). SAMe is believed to have a positive influence on multiple neuropsychiatric disorders and due to its role in increasing catechol-O-methyltransferase (COMT) activity, SAMe has been suggested to reduce aggressive behavior in psychiatric patients (45, 46).

Of the GABAergic metabolites, only succinic acid was included in the top 25% most strongly associated biomarker results. In the discovery and replication analyses succinic acid showed, non-significant, higher levels in children with high aggression, though the direction of effect flipped in the replication analysis. In contrast to our findings, succinic semialdehyde dehydrogenase (SSADH) deficiency, a rare inherited metabolic disorder, causes lower succinic acid levels and has been associated with a number of neuropsychiatric symptoms, including aggressive behavior (47). The top 25% did include other metabolites involved in the metabolism of GABAergic metabolites. For example, gamma-glutamylglutamine is a dipeptide obtained from glutamine and L-glutamic acid, low levels of gamma-glutamylglutamine reflect a deficiency in gamma-glutamyltransferase system responsible for glutamate transport across the membrane (48); congruent with a previous study in drug naive patients with schizophrenia, where lower levels of cerebrospinal fluid (CSF) were observed as compared to controls (49), we reported lower levels in children with high aggression. Furthermore, in the discovery analyses we observed significantly increased levels of gamma-L-glutamyl-L-alanine, after correction for multiple testing. Gamma-L-glutamyl-L-alanine is formed by the condensation of L-glutamic acid and L-alanine. Finally, we also observed dysregulation of metabolites downstream from GABAergic metabolites, such as L-arginine, which is synthesized from glutamine through citrulline. We observed lower levels of L-arginine in children with high aggression. Our results are consistent with results obtained for other psychiatric disorders, so have lower serum L-arginine levels been associated with antisocial personality disorder (APD) and schizophrenia (50, 51).

### Dysregulation in Oxidative Stress Pathways and Childhood Aggression

Inflammation has been identified as a potential mechanism underlying aggressive behavior (16, 52). One of the mechanisms believed to induce chronic inflammation is oxidative stress, characterized by the disturbed balance between antioxidant defenses and the production of reactive oxygen species (53). In the discovery analyses we reported significantly higher levels of the composite measure for oxidized DNA/RNA in children with high aggression, though in the replication analysis we observed non-significant lower oxidized DNA/RNA levels for children with high aggression. A study investigating the role of oxidative stress in adults with intermittent explosive disorder (IED) observed increased plasma levels of the oxidative stress markers 8-hydroxy-2′-deoxyguanosine and 8-isoprostane (54). Congruent with our results in the discovery analyses, Coccaro et al. (54) also reported significant positive correlation of oxidative stress markers with aggression.

In addition to dysregulation in oxidative stress markers, we have observed dysregulation in several metabolites involved in oxidative stress pathways. As discussed, we found lower levels of L-arginine in children with high aggression. L-arginine is synthesized from glutamine through citrulline. Both L-arginine and citrulline are precursors for nitric oxide, with low citrulline levels indicating overconsumption of citrulline for nitric oxide synthesis (55). Through nitric oxide mediation citrulline can play a role in oxidative stress.

Similarly, SAMe has been discussed for its role in norepinephrine metabolism. While SAMe was not measured in the current study, methionine sulfoxide was included in the top 25% most strongly associated biomarkers. Methionine sulfoxide is obtained by oxidation of the sulfur in methionine and high serum methionine levels have been associated with anger and indirect aggression in APD patients (50). In contrast, lower plasma methionine levels have been reported in MDD patients as compared to controls (40, 56). Furthermore, after correction for multiple testing higher levels of the methionine precursor, L-alpha-aminobutyric acid, were observed for children with high aggression. SAMe is also a precursor for the cysteine metabolism pathway, which is involved in the synthesis of the antioxidant glutathione (57). Low glutathione production might cause oxidative stress (53). Further suggestive evidence for a role of the cysteine metabolism pathway comes from the significant positive association of gamma-L-glutamyl-L-alanine with childhood aggression. Gamma-L-glutamyl-L-alanine is a gamma-glutamyl peptide and a substrate of a metabolite involved in glutathione metabolism. A study in mice showed that gamma-glutamyl peptides are synthesised through reactions with gamma-glutamylcysteine and glutathione synthetase and that this particularly occurs when glutathione is depleted (58). This is evident from the observation that elevated gamma-glutamyl peptide levels coincide with decreased glutathione levels in mice (58, 59). These findings suggest that increased levels of the gamma-glutamyl peptide, gamma-L-glutamyl-L-alanine, may reflect depleted glutathione levels and supports a role for oxidative stress in childhood aggression.

In general, inflammation and oxidative stress have been associated with a great number of neuropsychiatric disorders (60), therefore, it is likely that these mechanisms do not play a role in childhood aggression specifically, but might be more general mechanisms underlying neuropsychiatric disorders. However, knowledge of the causal mechanisms linking inflammation and oxidative stress with neuropsychiatric disorders is largely lacking.

### Energy Metabolism and Childhood Aggression

The results as obtained in the discovery replication and validation analyses also suggest a potential role of energy metabolism dysregulation in childhood aggression. Many of the main metabolic pathways are involved in converting glucose into energy (glycogenesis) and the breakdown of proteins to produce glucose (gluconeogenesis) to maintain blood glucose levels (61). We found L-aspartic acid, which is involved in gluconeogenesis to differ significantly between twins scoring high on aggression as compared to their low scoring co-twins in the within-family replication analyses. While we reported lower urinary L-aspartic acid levels in twins with high aggression, a previous study reported increased serum levels in patients with APD (50). Congruent with our findings, lower plasma levels of L-aspartic acid were reported in MDD patients as compared to controls (56). While glucose is the main energy source in the human body, in cells and tissues with high-energy demand, such as the skeletal muscles, the phosphorylation of creatine produces phosphocreatine, a major source for adenosine triphosphate [ATP; (62, 63)]. During the conversion of creatine to phosphocreatine, creatinine is formed spontaneously (62). We consistently, but not always significantly, report higher creatinine levels in children with high aggression as compared to children with low aggression across all three phases of the study. Plasma creatinine has been associated with the severity of depression symptoms (64) and patients with schizophrenia showed decreased blood creatinine levels as compared to controls (65). Processes for storing and obtaining energy in and from fatty molecules are related to energy metabolism. In the current study the current study we find associations with childhood aggression for ethanolamine, involved in the synthesis of phospholipids, N6-N6-N6-trimethyl-L-lysine, involved in oxidation of fatty acids, and glyceric acid, involved in glycerolipid metabolism. Previously, glyceric acid was included in a panel capable of discriminating between patients with schizophrenia and controls with an AUC of 0.94 (66), lower serum levels of ethanolamine were observed in APD patients as compared to controls (50), lower CSF levels of ethanolamine were reported in MDD patients as compared to controls and associated to depression severity and increased somatic anxiety symptoms in MDD patients (67), in addition, serum levels of N6-N6-N6-trimethyl-L-lysine have been associated with cognitive decline (68).

### Strengths and Limitations

This study has several assets. First of all, the large-scale study design, which could be achieved by investigating urinary biomarkers, is a major strength of the current study. Urine is an easily accessible biofluid and may be obtained with minimal invasiveness, making it an ideal measure for large-scale data collection in vulnerable groups, like children. We showed that large scale standardized collection of urine and buccal samples is feasible in epidemiological projects and attained a fairly high response rates, considering that the sample included families who had to cope with difficult children. Families successfully kept samples at home in their freezers, until transport to the laboratory. Obviously, collection of frozen samples from a population-based sample at home is only feasible in a small country like the Netherlands.

The use of a longitudinal twin cohort permitted us to select children that were stable in their aggression status over time (see Supplementary Table 5). We have shown that the operationalization of high and low aggression in our twin sample on the basis of previously collected data across ages, raters, and instruments did not impact mean aggression differences between concordant and discordant twin pairs at urine collection. By including twin pairs who were concordant (high-high or low-low) in their aggression scores, we further optimized toward more extreme groups. The MZ twin pairs discordant for aggression, enabled the analysis of within-family differences and controlled for genetic differences between individuals as well as potential confounders from the shared home or school environment, as these are largely shared between MZ twins. Finally, the clinical cases as included in the validation sample had aggression scores at the extreme end of the aggression distribution. As such, differences reported in the validation analyses between clinical cases and twin controls, are likely to offer the best indication of dysfunctional aggression. However, it should also be noted that the Aggressive Behavior subscale of the CBCL is derived from data-driven, factor analytic approaches. Consequently, the scale includes several items that wouldn't be considered aggressive based on their content (e.g., Unusually loud, Sulks). Therefore, approaches with more theory-driven definitions of aggression (e.g., predatory aggression) should also be explored.

Our collection protocol was tested extensively, and it was kept relatively simple to ensure compliance. As a consequence, the collected first-morning urine was not mid-stream, as is sometimes recommended to avoid potential bacterial contamination of the upper urinary tract (69). Fortunately, dipstick results for the urine samples did not indicate serious contaminations (data not shown), indicating that these did not play a major role in our findings. Because urine collection was performed by parents and children in the home-setting, deviations from the collection protocol were poorly monitored. Future studies may consider pairing the urine collection brochures with short videos describing the protocol to make it more accessible. Integrating such videos in an app, together with the phenotypic data collection can allow for the monitoring of the collection protocol and may also increase protocol compliance.

In interpreting our results, the wide age range (5–13 years of age) included in our study should be considered. This is because the onset of puberty likely influences both aggressive behavior and urinary metabolite profiles in older children. A caveat of the analyses targeting neurotransmitter ratios is the inability of targeting the complete neurotransmitter pathways. Our platforms did not target 5-hydroxyindoleacetic acid (5-HIAA), succinate semialdehyde, levodopa, dopamine, or 3,4-dihydroxyphenyl acetic acid (DOPAC). Moreover, the relationship of urine and brain metabolites is poorly understood, as many of our metabolites of interest are also synthesized in peripheral systems, therefore urinary metabolites do not necessarily reflect processes in the brain (70). Finally, in addition to all item-specific analyses, the results for 19 metabolites in general must be interpreted with caution because their RSDqc values fell outside of the acceptable range (>15%), this includes gamma-L-glutamyl-L-alanine, which was included in the top 25% most associated metabolites.

## Conclusions and Future Directions

This was the first metabolomics study on childhood aggression. In both the discovery and replication phases of this study we reported metabolites significantly associated with childhood aggression, however, these results were not congruent between the analyses and could not be validated. Our top metabolites play roles in central metabolic processes, specifically energy metabolism, neurotransmission, and oxidative stress. While most of the metabolites have previously been associated with neuropsychiatric disorders, only L-tryptophan and oxidized DNA/RNA are known to be involved in adult aggression. Further work is required to replicate our results and to establish the viability of the suggested urinary biomarkers in the early detection or treatment of childhood aggression, as the translational applicability for the current results are still limited. For a biomarker panel to be of practical utility it needs to exhibit good discrimination among phenotype classes, with high specificity and sensitivity (2). The metabolite levels analyzed were quantified relative to an internal standard. To develop a biomarker panel with practical utility and recommended threshold values, absolute quantified values are preferred.

Moreover, while this study described the associations for a large number of amines and organic acids, it has not included the contribution of steroid hormones, as well as their interaction with neurotransmitters. This is an active topic in aggression research and in our ACTION project we aim to include the measurement of steroid hormones. Elucidating the role of steroid hormones, particularly in conjunction with metabolomics, may be of benefit to the field. Finally, all current results are correlational, therefore considerably more work needs to be done to determine the causal role of metabolic dysregulation in (childhood) aggression, combining multiple types of ‘omics techniques (e.g., genomics, epigenomics, metabolomics) could be of aid here.

## Data Availability Statement

We developed a standardized protocol for the large-scale collection of urine samples in children, which has been made available to the scientific community (http://www.action-euproject.eu/content/data-protocols).

## Ethics Statement

The studies involving human participants were reviewed and approved by the Central Ethics Committee on Research Involving Human Subjects of the VU University Medical Center and ethics board of Leiden University Medical Center. Written informed consent to participate in this study was provided by the participants' legal guardian/next of kin.

## Author Contributions

CK, AH, TH, VF, MB, RV, and DB contributed to the conception and design of the study. FH, MV, and PR were responsible for biological sample collection. CB and EZ were responsible for phenotypic data collection, cleaning, and preprocessing in the Netherlands Twin Register. CK, ST, AH, and TH were responsible for biomarker and metabolomics data acquisition. CK, AH, RP, and FH performed the cleaning and preprocessing of the biomarker and metabolomics data. EE and GD were responsible for genotyping. EE, GD, JH, and JD were responsible for the cleaning and preprocessing of the (epi)genetic data. FH, PR, and SD performed the statistical analysis. FH, PR, and RP created the figures and tables. FH and PR wrote the first draft of the manuscript. RP, AH, ST, and DB wrote sections of the manuscript. All authors contributed to manuscript revision, read, and approved the submitted version.

### Conflict of Interest

CK and ST were employed by Good Biomarker Sciences (Leiden, the Netherlands). EE and GD were employed by the Avera Institute for Human Genetics (Sioux Falls, SD, United States). The remaining authors declare that the research was conducted in the absence of any commercial or financial relationships that could be construed as a potential conflict of interest.
