# Supplementary material for: Urinary Amine and Organic Acid Metabolites Evaluated as Markers for Childhood Aggression: The ACTION Biomarker Study
Source: Front Psychiatry. 2020 Mar 31;11:165. doi: 10.3389/fpsyt.2020.00165 (PMC7138132; doi:10.3389/fpsyt.2020.00165)
Supplement: Supplementary file 2 [file Data_Sheet_2.docx]

Supplementary Material

# Supplementary Text 1: brief description of buccal sample collection for (epi)genetics in ACTION in the Netherlands Twin Register

Buccal cells for DNA isolation and genotyping were collected during two days and were also collected from parents and additional siblings. All parents provided written informed consents for their own and their children’s participation. Genotyping was done on the Axiom (*N* = 861; Ehli et al. 2017) or the GSA array (*N* = 2,151; Beck et al. 2019). Genotyping data were analyzed to establish zygosity (Odintsova et al. 2019), of which parents received the results.

For epigenetics 108 extra twins with buccal-cell samples and longitudinal aggression data were included from the NTR database. Thus in total 1,475 twins (737 complete pairs), either with first-morning urine (*N* = 1,362) and/or buccal-cell swabs (*N* = 1,468), were included in the ACTION project (**Supplementary Table 1**). In the twins, epigenetic markers were measured on the Illumina EPIC 850K array (Van Dongen et al. 2018).

# Supplementary Text 2: medication use and other covariates

In the sensitivity analyses we assessed the potential impact of preexisting chronic conditions, medication use, or vitamin use on differences in biomarker levels and neurotransmitter ratios between the MZ twins scoring high and low on aggression.

Medication use has been assessed in the twin cohort through parent report at the time of urine collection, in the clinical cohort medication use was extracted from the patient files. ATC codes (<https://www.whocc.no/atc_ddd_index/>) were assigned to the medications used at the time of urine collection in both cohorts. Based on the ATC codes medications could be classified. At time of urine collection children included in the current study used medications classified as: alimentary tract and metabolism (A), cardiovascular system (C), dermatological (D), genito-urinary system and sex hormone (G), systemic hormonal preparations (H), anti-infectives for systemic use (J), nervous system (N) and respiratory system (R) medications.

Children were most frequently using nervous system or respiratory system medications, which is consistent with reported incidences of asthma or allergies. The respiratory medications included nasal preparations (R01), drugs for obstructive airway diseases (R03) and antihistamines for system use (R06). The nervous system medications included analgesics (N02, e.g., paracetamol use), antiepileptics (N03), psycholeptics (N05), psychoanaleptics (N06) and other nervous system drugs (N07; here chiefly antivertigo medications). Medications belonging to the N05 and N06 classes (e.g., aripiprazole [N05AX12] or methylphenidate [N06BA04]) are also considered psychotropic medications and are prescribed for the treatment of psychiatric disorders, including for example attention-deficit/hyperactivity disorder. In **Table 1** we included an overview of the number of children on psychotropic medications in both cohorts.

# Supplementary Text 3: Aggressive Behavior item-based biomarker discovery

## Assessment of aggressive behavior

At or near the time of biological sample collection parents completed the CBCL. The CBCL Aggressive Behavior subscale consists of 18 items assessing multiple aspects of aggressive behavior (see **Supplementary Table 4**). Parents were asked to indicate the applicability of each item to their child’s behavior over the past 6 months. Answer categories ranged from “not true” (coded as “0”), to “somewhat or sometimes true” (coded as “1”), and “very true or often true” (codes as “2”). All items were dichotomized to reflect case/control status, with items scored as “not true” defining control status. The answer categories “somewhat or sometimes true” and “very true or often true” both reflected case status. Endorsement of the original answer categories as well as the dichotomized answer categories have been supplied in **Supplementary Table 4**. In the NTR, items from mother-rated CBCL Aggressive Behavior subscale were analyzed, in the Curium-LUMC cohort the majority (90%) of ratings was also by the mother.

## Statistical analyses

In the item-based discovery, replication and validation phases the same subjects as in the original discovery, replication and validation phases were classified as cases or controls based on each of the 18 CBCL Aggressive Behavior items (see **section 3.1**). GEE analyses, including sex and age as covariates, assessed the relationship of the biomarkers and neurotransmitter ratios with item case-control status. Analyses were corrected for relatedness using an ‘exchangeable’ correlation structure. The FDR of 5% at a threshold of p ≤ 0.05 for 1602 tests (biomarkers) or 126 tests (ratios) are provided. Note, that because of the large number of tests interpretation of the discovery phase in terms of significance is complex. The top 25% most associated biomarkers or ratios per item were tested in the replication phase. In the replication phase the FDR of 5% for 414 tests (biomarkers) and 54 tests (ratios) at p ≤ 0.05 was used. Finally, in the validation phase the biomarkers or ratios with congruent directions of effect in the discovery and validation phase and which were significantly associated with item case-control status in the validation phase were assessed. For those items without significantly associated biomarkers or ratios, the top 5 biomarkers or top ratio were assessed in the replication phase. The significance threshold was set at p ≤ 0.05 with a 5% FDR for 88 tests (biomarkers) and 18 tests (ratios) to control multiple testing.

## Results

### Participant descriptives

Both the original and dichotomized responses for each of the 18 items have been included in **Supplementary Table 4**. Case and control status on an item-to-item basis vary considerably across children (**Supplementary Table 4**). It must be noted that for some items, particularly the more extreme items such as “Threatens other people”, item endorsement is low across all groups (**Supplementary Table 4**). As a consequence, meaningful interpretation of associated metabolites, other biomarkers and neurotransmitters is not always feasible.

### Association of urinary metabolites and other biomarkers with Aggressive Behavior items

#### Discovery

The discovery analyses showed significant metabolites or other biomarkers for each of the 18 Aggressive Behavior items, overall 3.8% of the tests were significant, however, after correcting for multiple testing none of the item-specific metabolites or other biomarkers remained significant (**Supplementary Table 13**). Comparing the top 25% metabolites and other biomarkers for overall aggression, we observe that of the 23 metabolites or other biomarkers in the top 25% between 2 and 12 overlap per item (**Supplementary Table 14**). Of the overlapping metabolites or other biomarkers approximately 78% have congruent directions of effect among the overall aggression and item-specific analyses (**Supplementary Table 14**).

#### Replication

The top 25% most associated metabolites and other biomarkers per item were assessed for replication in a sample of twin pairs discordant for aggression. In the replication analyses 29 metabolites or other biomarkers were significantly associated with aggression items, here only 12 of the 18 aggression items had significantly associated metabolites or other biomarkers (**Supplementary Table 15**). In total 8.5% of the total number of conducted tests were significant. Five of the significantly associated metabolites or other biomarkers were also included in the top 25% for overall aggression. In the replication analyses isocitrate was associated with ‘Disobedient at home’ (*β* = 0.26; *SE* = 0.10; *p* = 0.008), for overall aggression this metabolite was not significant and showed an opposite direction of effect in the replication analysis. ‘Disobedient at home’ was also significantly associated with norepinephrine levels (*β* = 0.22; *SE* = 0.10; *p* = 0.03), in the overall aggression replication analysis this metabolite was also significantly associated, before multiple testing correction; however the association was in the opposite direction (mean difference = -0.19; *p* = 0.02). The associations of ethanolamine with ‘Disobedient at school’, isocitrate with ‘Threatens’ and succinic acid with ‘Temper’ were in the same direction of effect as observed for overall aggression (**Supplementary Table 7** and **15**). Only ethanolamine was significantly associated with both ‘Disobedient at school’ (*β* = -0.31; *SE* = 0.14; *p* = 0.03) and with overall aggression (mean difference = -0.20; *p* = 0.03). After correction for multiple testing 10 of the 15 (66.7%) metabolites or other biomarkers associated with ‘Threatens’ were still significant (**Supplementary Table 15**). However only 3 children were cases for ‘threaten other people’ (**Supplementary Table 4**). For the other 11 items none of the metabolites or other biomarkers remained significant after correction for multiple testing (**Supplementary Table 15**). Overall, we observed congruent directions of effect in the discovery and validation analyses for 3-19 out of 23 (13.0-82.6%) top 25% amines, organic acids and biomarkers per item (**Supplementary Table 14**).

#### Validation

For the validation analyses we selected the top 5 most associated metabolites or other biomarkers from the replication analyses with congruent directions of effects in the discovery analyses. For the ‘Fights’ item only 3 metabolites or other biomarkers showed congruent direction of effect between the discovery and the replication, therefore, only these 3 were included. In the validation analyses neopterin is significantly associated with ‘Argues’ (*β* = -0.25; *SE* = 0.10; *p* = 0.01) and L-proline with ‘Mean’ (*β* = -0.28; *SE* = 0.12; *p* = 0.02). None of the other biomarker-item combinations were significant and after correction for multiple testing, the associations of neopterin with ’Argues’ and L-proline with ‘Mean’ were no longer significant (**Supplementary Table 16**). Overall, congruent directions of effect between the replication and validation were observed for 0-4 out of the top 5 (0%-80%) amines, organic acids and biomarkers per item (**Supplementary Table 14**).

### Association of urinary neurotransmitter pathways with Aggressive Behavior items

#### Discovery

To elucidate the role of serotonergic, dopaminergic and GABAergic neurotransmitter pathways we performed discovery analyses with gee analyses for each of the 18 items of the CBCL Aggressive Behavior subscale. The discovery analyses showed that the catabolic dopamine neurotransmitter ratio 3MT to HVA was significantly associated with the ‘Stubborn’ (*β* = -2.50; *SE* = 1.16; *p* = 0.03) and ‘Sulks’ (*β* = -3.17; *SE* = 1.59; *p* = 0.05) items. The catabolic GABA neurotransmitter ratio GABA to succinic acid was significantly associated with the ‘Physically attacks people’ (*β* = -2.49; *SE* = 0.90; *p* = 0.01), ‘Suspicious’ (*β* = -1.82; *SE* = 0.83; *p* = 0.03) and ‘Teases’ (*β* = -2.34; *SE* = 0.88; *p* = 0.01) items. The anabolic GABA neurotransmitter L-glutamic acid to GABA was significantly associated with ‘Disobedient at School’ (*β* = -3.34; *SE* = 1.62; *p* = 0.04). After correction for multiple testing none of the neurotransmitter ratio-item associations was significant and none of the neurotransmitter ratios involved in the anabolism or catabolism of serotonin, dopamine or GABA significantly associated with the other 12 aggressive behavior items (**Supplementary Table 17**). None of the most associated neurotransmitter ratios per item were included in the top 25% most associated neurotransmitter items for overall aggression. Of the 7 neurotransmitter ratios congruent directions of effect between the overall aggression discovery results and the item specific results were observed for 6 ratios across 17 items, with no congruent directions of effect observed for the ratio of 5HTP to serotonin and for the ‘Sudden changes in mood or feelings’ item (**Supplementary Table 18**).

#### Replication

Replication of the top 25% most associated neurotransmitter ratios from the item-specific discovery analyses were performed in the sample of twins discordant for overall aggression. The anabolic dopamine ratio L-phenylalanine to L-tyrosine was significantly associated with the ‘Fights’ (*β* = -0.85; *SE* = 0.42; *p* = 0.04) and ‘Threatens’ (*β* = -1.19; *SE* = 0.39; *p* = 0.002) items, though these associations did not survive multiple testing (**Supplementary Table 19**). The direction of effect of L-phenylalanine to L-tyrosine for the ‘Fights’ and ‘Threatens’ items were congruent with the direction of effect as observed in the discovery analyses (**Supplementary Table 18**). None of the other neurotransmitter ratio aggression item combinations reached significance in the replication analyses (**Supplementary Table 19**) and the congruence of effect directions ranged from none (‘Mean’) to all (3; ‘Threatens’), with an average of 1.5 (**Supplementary Table 18**)

#### Validation

The top neurotransmitter ratio for each item was assessed in a sample of clinical cases and twin controls. Before correction for multiple testing the anabolic dopamine neurotransmitter ratio L-phenylalanine to L-tyrosine was significantly associated with the ‘Disobedient at school’ (*β* = 4.64; *SE* =1.96; *p* = 0.02) and ‘Loud’ (*β* = 4.18; *SE* = 2.00; *p* = 0.04) items (**Supplementary Table 20**). For ‘Disobedient at school’ the direction of effect has flipped as compared to the replication analysis, for ‘Loud’ the direction of effect was congruent across the replication and validation phases (**Supplementary Table 18**). Neurotransmitter ratios were not significantly associated with any of the other 16 aggression items and after correction for multiple testing the ratio of L-phenylalanine to L-tyrosine was not significantly associated with ‘Disobedient at school’ or ‘Loud’ (**Supplementary Table 20**). In addition to the congruent direction of effect for ‘Loud’ we also observed congruent directions of effect of ‘MoodSwings’, ‘Suspicious’ and ‘Teases’ (**Supplementary Table 18**).

# Supplementary Text 4: description of aggression measures

In **Supplementary Table 5** we present the mean scores of the twins included in this project for aggression as obtained by different raters and instruments at different ages. The following questionnaires have been included in this overview:

- The Aggressive Behavior scale of the ASEBA Child Behavior Checklist (CBCL) for preschool children (1.5-5 years; Achenbach, Ivanova, and Rescorla 2017) as rated by mothers and fathers of the twins at age 3.
- The Aggressive Behavior scale of the Devereux Child Behavior (DCB) rating scale (van Beijsterveldt et al. 2004; Molenaar et al. 2015) as rated by mothers and fathers of the twins at age 5.
- The Aggressive Behavior scale of the ASEBA CBCL for school-aged children (6-18 years; Achenbach, Ivanova, and Rescorla 2017) as rated by mothers and fathers of the twins at ages 7 and 10.
- The Aggressive Behavior scale of the ASEBA Teacher Rating Form (TRF; Achenbach, Ivanova, and Rescorla 2017) as rated by teachers of the twins at ages 7, 10 and 12.
- The Conduct Problems scale of the Strengths and Difficulties Questionnaire (SDQ; Goodman 1997, 2001) as rated by mothers and fathers of the twins at age 10.
- The Conduct Disorder (CD) and Oppositional Defiant Disorder (ODD) scales from the Autism - Tics, ADHD and other Comorbidities inventory (A-TAC; Hansson et al. 2005; Kerekes et al. 2014) as rated by mothers and fathers of the twins at age 10.
- The Aggressive Behavior scale of the ASEBA Brief Problem Monitor (BPM; Chorpita et al. 2010) as rated by mothers and father of the twins at age 12.

# References

Achenbach, Thomas M., Masha Y. Ivanova, and Leslie A. Rescorla. 2017. “Empirically Based Assessment and Taxonomy of Psychopathology for Ages 1½–90+ Years: Developmental, Multi-Informant, and Multicultural Findings.” *Comprehensive Psychiatry* 79: 4–18. https://doi.org/10.1016/j.comppsych.2017.03.006.

Beck, Jeffrey J., Jouke-Jan Hottenga, Hamdi Mbarek, Casey T. Finnicum, Erik A. Ehli, Yoon-Mi Hur, Nicholas G. Martin, Eco J.C. de Geus, Dorret I. Boomsma, and Gareth E. Davies. 2019. “Genetic Similarity Assessment of Twin-Family Populations by Custom-Designed Genotyping Array.” *Twin Research and Human Genetics*, August, 1–10. https://doi.org/10.1017/thg.2019.41.

Beijsterveldt, C E M van, F. C. Verhulst, P. C.M. Molenaar, and D. I. Boomsma. 2004. “The Genetic Basis of Problem Behavior in 5-Year-Old Dutch Twin Pairs.” *Behavior Genetics* 34 (3): 229–42. https://doi.org/10.1023/B:BEGE.0000017869.30151.fd.

Chorpita, Bruce F., Steven Reise, John R. Weisz, Kathleen Grubbs, Kimberly D. Becker, and Jennifer L. Krull. 2010. “Evaluation of the Brief Problem Checklist: Child and Caregiver Interviews to Measure Clinical Progress.” *Journal of Consulting and Clinical Psychology* 78 (4): 526–36. https://doi.org/10.1037/a0019602.

Dongen, Jenny Van, Erik A. Ehli, Rick Jansen, Catharina E.M. Van Beijsterveldt, Gonneke Willemsen, Jouke J. Hottenga, Noah A. Kallsen, et al. 2018. “Genome-Wide Analysis of DNA Methylation in Buccal Cells: A Study of Monozygotic Twins and MQTLs.” *Epigenetics and Chromatin* 11 (1): 1–14. https://doi.org/10.1186/s13072-018-0225-x.

Ehli, Erik A., Abdel Abdellaoui, Iryna O. Fedko, Charlie Grieser, Sahar Nohzadeh-Malakshah, Gonneke Willemsen, Eco J.C. De Geus, Dorret I. Boomsma, Gareth E. Davies, and Jouke J. Hottenga. 2017. “A Method to Customize Population-Specific Arrays for Genome-Wide Association Testing.” *European Journal of Human Genetics* 25 (2): 267–70. https://doi.org/10.1038/ejhg.2016.152.

Goodman. 1997. “The Strengths and Difficulties Questionnaire: A Research Note.” *J. Child Psychol. Psychiat* 38 (5): 581–86. https://journals-scholarsportal-info.proxy1.lib.uwo.ca/pdf/00219630/v38i0005/581_tsadqarn.xml.

Goodman, R. 2001. “Psychometric Properties of the Strengths and Difficulties Questionnaire.” *Journal of the American Academy of Child and Adolescent Psychiatry* 40 (11): 1337–45. https://doi.org/10.1097/00004583-200111000-00015.

Hansson, Sara Lima, Annika Svanström Röjvall, Maria Rastam, Carina Gillberg, Christopher Gillberg, and Henrik Anckarsäter. 2005. “Psychiatric Telephone Interview with Parents for Screening of Childhood Autism - Tics, Attention-Deficit Hyperactivity Disorder and Other Comorbidities (A-TAC): Preliminary Reliability and Validity.” *British Journal of Psychiatry* 187 (SEPT.): 262–67. https://doi.org/10.1192/bjp.187.3.262.

Kerekes, Nóra, Sebastian Lundström, Zheng Chang, Armin Tajnia, Patrick Jern, Paul Lichtenstein, Thomas Nilsson, and Henrik Anckarsäter. 2014. “Oppositional Defiant- and Conduct Disorder-like Problems: Neurodevelopmental Predictors and Genetic Background in Boys and Girls, in a Nationwide Twin Study.” *PeerJ* 2: e359. https://doi.org/10.7717/peerj.359.

Molenaar, Dylan, Christel Middeldorp, Toos van Beijsterveldt, and Dorret I. Boomsma. 2015. “Analysis of Behavioral and Emotional Problems in Children Highlights the Role of Genotype × Environment Interaction.” *Child Development* 86 (6): 1999–2016. https://doi.org/10.1111/cdev.12451.

Odintsova, Veronika V., Peter J. Roetman, Hill F Ip, René Pool, Camiel M. Van der Laan, Klodiana-Daphne Tona, Robert R.J.M. Vermeiren, and Dorret I. Boomsma. 2019. “Genomics of Human Aggression.” *Psychiatric Genetics* 29 (5): 170–90. https://doi.org/10.1097/YPG.0000000000000239.
